# Data on artificial recharge sites identified by geospatial tools in semi-arid region of Anantapur District, Andhra Pradesh, India

**DOI:** 10.1016/j.dib.2018.04.050

**Published:** 2018-04-21

**Authors:** M. Rajasekhar, G. Sudarsana Raju, R. Siddi Raju, U. Imran Basha

**Affiliations:** a Dept. of Geology, Yogi Vemana University, Kadapa, Andhra Pradesh, India; b Dept. of Geology, Sri Venkateswara University, Tirupathi, Andhra Pradesh, India

**Keywords:** Geospatial technologies, MIF, AHP, Overlay analysis, Thematic maps

## Abstract

The Geospatial Technologies like Remote Sensing (RS) and Geographic Information System (GIS) have been playing vital role in capable forecasting and management of imperative groundwater resources in the emerging nations. In recent times, the geospatial technologies like RS, GIS and Multi Influence Factor (MIF) methodology are helpful in identifying groundwater potential zone. For the present study, the geospatial technology is used to prepare various thematic maps such as Land Slope, Geomorphology, Geology, Soil, Drainage Density, Lineament Density, Landuse/Landcover, Hydrogeomorphology, and Annual consideration of the valuation of groundwater assets for the semi-arid region in and around Bommanahal Mandal of Anantapur District in Andhra Pradesh, Southern India. As a part of the study eight thematic layers and their functions have been designed applicable weights at the Saaty׳s scale according to their comparative connotation in groundwater occurrence. The designed weights are normalized by using AHP (Analytic Hierarchy Process) MIF techniques and eigenvector method to various thematic layers and their features. Further to create a groundwater potential map the chosen thematic maps are integrated by weighted linear grouping method in a GIS environment. Based on the groundwater potential index values, the study area is classified into four different groundwater potential zones such as ‘good’, ‘moderate to good’, ‘moderate’ and ‘poor’. The new recharge structures have proposed to fulfill the demand of groundwater to expand the scope of groundwater for future generations. Considering the overlay analysis of geomorphology and drainage layer execution through GIS technologies, the appropriate sites for artificial recharge structures have been identified.

**Specifications table**

**Table:** 

*Subject area*	*Hydrogeology and Remote Sensing & GIS*
*More specific subject area*	*Remote Sensing and GIS*
*Type of data*	*Table, Geospatial data and SRTM DEM*
*How data was acquired*	*Toposheets from Survey of India, Landsat 8 Satellite Imageries from USGS website and Field surveys*
*Data format*	*Processed and Analyzed*
*Experimental factors*	*Toposheets and Satellite imageries are georeferenced and digitized by using ArcGIS 10.4 & ERADAS imagine software.*
*Experimental features*	*Artificial Recharge structures are suggested to implement of groundwater management for future generation.*
*Data source location*	*76*° *51׳ 30′′ to 77*° *8׳ 30׳׳ N and 14*° *52׳ 0׳׳ to 15*° *25׳ 30׳׳ E*
*Data accessibility*	*The data are available with this article.*

**Value of the data**

• It can serve as baseline data for the artificial recharge structures construction of the area.
• Data presented can be utilized for the execution of ground water recharge and management.
• Data are georeferenced and digitized, for the future studies.
• It is also resourceful to researchers, stakeholders, hydrogeologists for aquifer management.
• It can enhance the socio-economic status of the study area.

## 1 Data

The data described as geospatial technologies is precise, concise and repetitive analysis of the earth׳s features in different bands of the electromagnetic spectrum. Geospatial technology offers vast areas of exact spatial and temporal frequencies. It finds extensive applications in including hydrological studies such as watershed management, identification of artificial recharge structures and hydrological modeling etc. It is also considered as a capable time. When it is compared and analyzed with the conventional methods of hydrological survey remotely sensed data are usually cost-effective inaccessible areas takes time for management exclusively be of massive significance. The different thematic maps such as geological geomorphological, soil, slope, landuse/cover, drainage, lineament etc delivers various information about factors that are controlling directly or indirectly in the occurrence by groundwater migration of groundwater by using Remote Sensing as well as GIS [1]. Further to handle the large and complex geographical data and to manage groundwater resources GIS perform key main role in geospatial technologies [2], [3], [4], [5], [6]. These thematic maps are then coordinated in a GIS system to distinguish reasonable zones for artificial recharge.

A couple of researchers have endeavored to choose suitable regions for artificial recharge and also to recommend remarkable recharge structures [7], [8]. Each artificial recharge procedure has its own particular attributes and the strategy for site assurance will contrast for each technique. Recharge basin is designed in moderate to good permeable regions, and this strategy is most appropriate in the study region due to its high practicability, proficiency and easy maintenance. For the study, site selection for artificial recharge is considered in semi-regions in parts of Anantapur region of Andhra Pradesh.

### 1.1 Study area

**Fig. 1:**
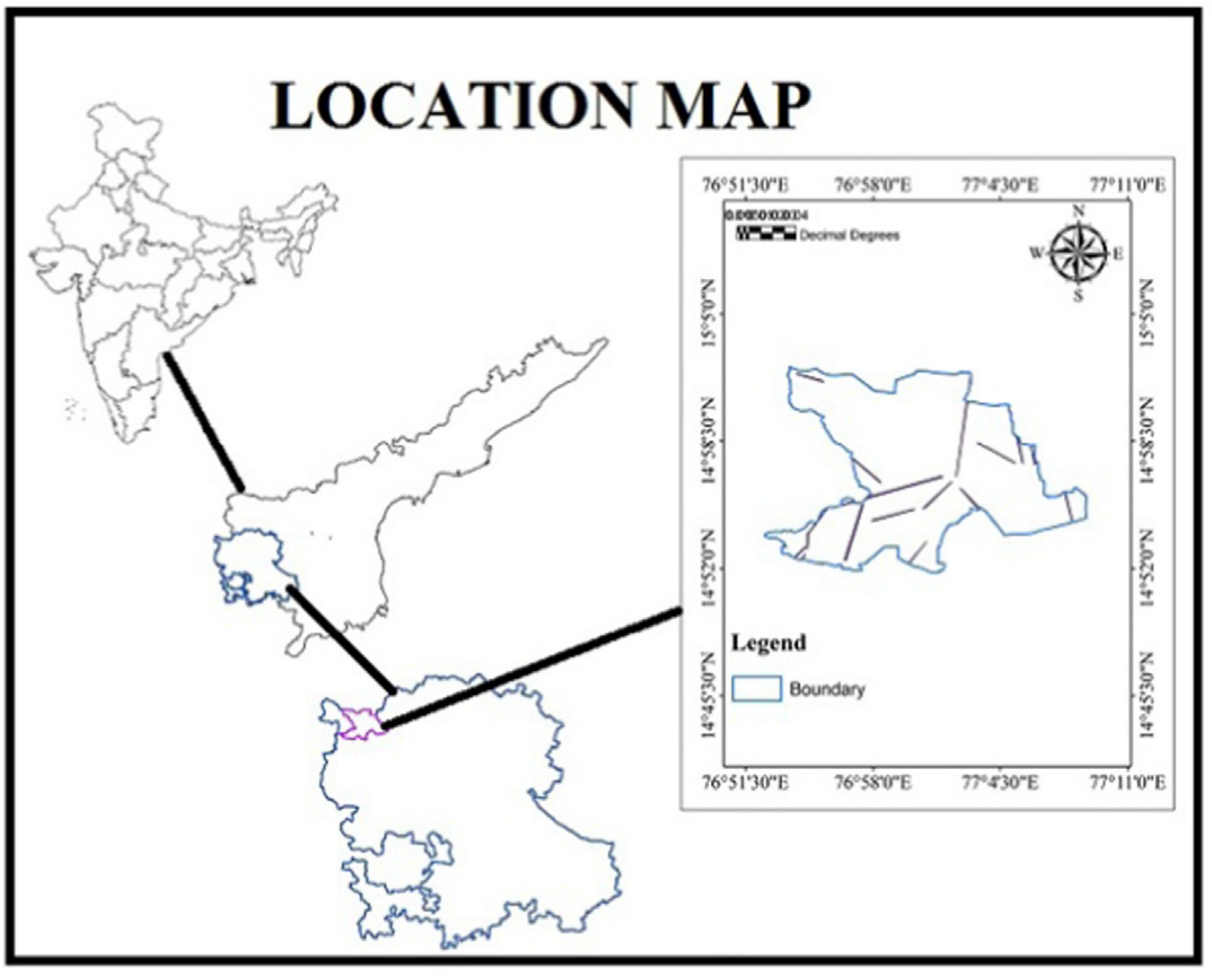
Location map of the study area.

The study area is located in the Survey of India Toposheet Nos: 57A/16, 57 B/13, 57 E/4 and 57 F/1 on 1:50,000 scale and lies between North longitudes 76° 51׳ 30’’ to 77° 8׳ 30׳׳ and East latitudes 14° 52׳ 0׳׳ to 15° 25׳ 30׳׳ (Fig. 1).It comprises a total geographical area of 305.88 sq km. A popular investigation soil survey of Anantapur district exhibits the occurrence of exclusive soil sequence and their associations in the study area. The soil sequence can be largely classified into three categories such as aridsols, alfisols and vertisol (National Bureau of Soil Survey and Land Utilization planning (NBSSLUP)). The study region is occupied wide vary of high grade metamorphic rocks of Hornblende-Gneiss/Hornblede-Biotite Gneiss. These rocks are substantially weathered and overlain with the aid of recent fills and alluvial at places. The major rock types existing in the river basin are felsic rock, pink granite and gneiss, Quartzite: BIF/BMQ ferruginous quartzite. The Vedavathi River is a predominant supply for irrigation, drinking water and for other human needs and necessities that are living on the river beds. The groundwater table drastically goes down due to indiscriminately sinking deep bore wells and negative recharge due to scanty rain and the absence of any surface water bodies.

## 2 Experimental design, materials and methods

In order to identify artificial recharge constructions in the study area, multi-parametric dataset comprising satellite data information and topographical data maps which include SOI toposheets were used. The base map of the study area is prepared by using SOI toposheets bearing no 57A/16, 57 B/13, 57 E/4, and 57F/1 in 1:50,000 scale. Land-use/Landcover map is prepared for the year 2012 from satellite images by using supervised and unsupervised classification using ERDAS Imagine 9.3 software. The ArcGIS 10.4 software used to prepare the distinctive thematic layers like geomorphology; soil, geology, and lineament density are prepared. To prepare the drainage density map of the study area, primarily, the drainage network for the study region was digitized from the SOI toposheets in 1:50,000 scale.

**Fig. 2:**
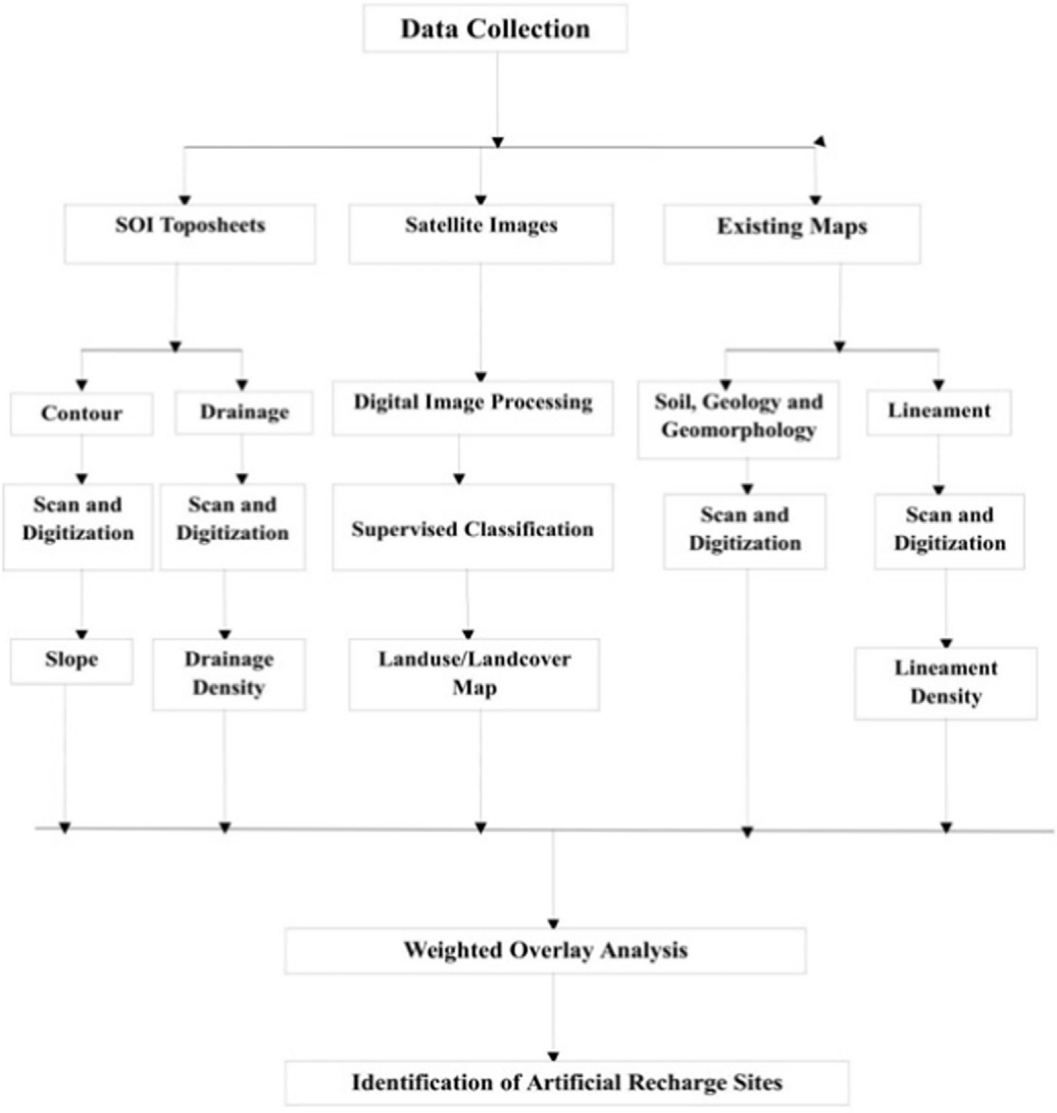
Methodology for identifying the artificial recharge site.

Further, in order to calculate drainage density, every drainage is expressed in terms of length of channels per unit vicinity (km/km^2^). After obtaining drainage densities, the total area was demarcated into advisable drainage density zones. For the preparation the slope map, elevation contours (10 m interval) had been digitized from the SOI (Survey of India) toposheets, and a digital elevation model (DEM) of the study region is generated. The Slopes are calculated from the elevation contours, and then a slope map used to be prepared by using ArcGIS 10.4 software. After preparing all the thematic layers, one of its features/classes of the individual themes are identified, which have been then assigned weights in accordance to their relative significance towards groundwater recharge in the study region. As mentioned above the eight thematic layers, viz., Land slope, Geomorphology, Geology, Soil, Drainage, drainage density, Landuse /land cover, Lineament density, Hydrogeomorphology are viewed for the delineation of artificial recharge zones. Two Thematic layers for these parameters has been prepared, classified, weighted and built-in by weighted overlay analysis. In Fig. 2 shows the overall methodology for identifying the artificial recharge structures.

### 2.1 Drainage

**Fig. 3:**
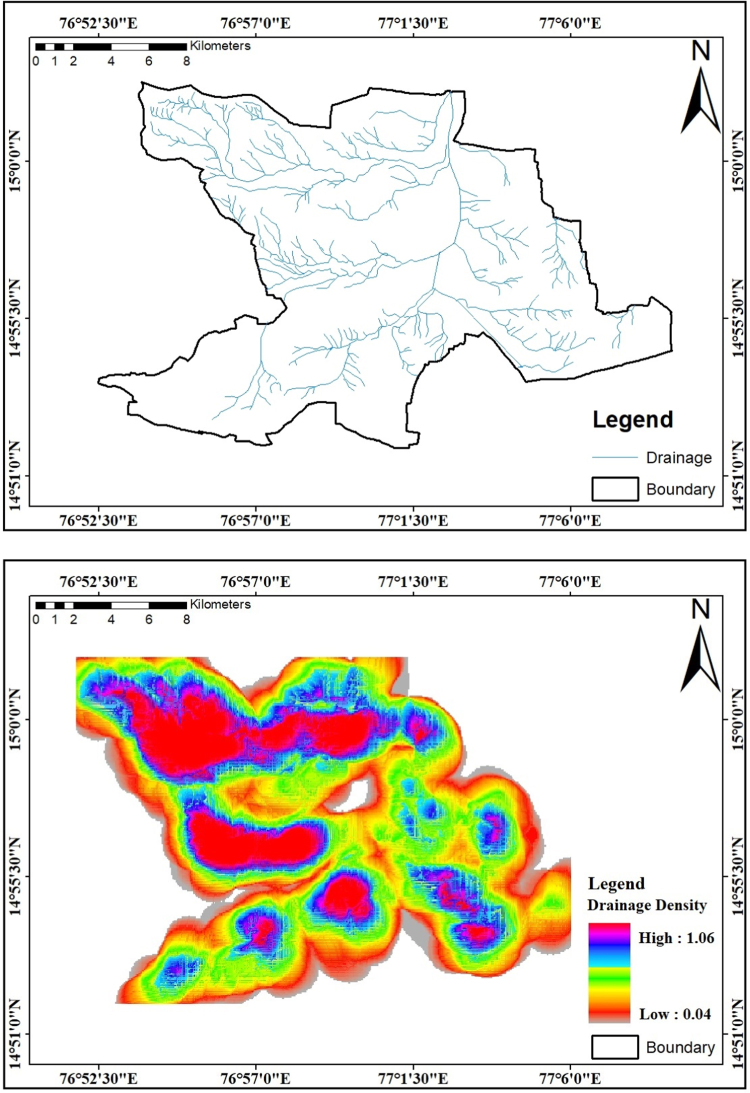
Drainage and density map of the study area in km/km^2^.

Drainage pattern reflects the aspect of surface to subsurface formation. Drainage pattern displays the element of surface to subsurface formation. Drainage pattern, texture and density are associated with runoff and vegetation which oscillate with climate. Relief and channel concavity play an important position as well as underlying lithology [9]. considering to the drainage pattern, drainage density map is prepared from the topographical map. It is separated into square grids of 1 Sq km and the whole lengths of all streams calculated in each grid and outcome the drainage density values in km/km^2^ shown in Fig. 3. The study region is primarily covered with dendritic drainage pattern having a high-quality drainage density in the southern phase of the study area, medium at the Centre and coarse drainage density is on the western aspect of the study region basin.

### 2.2 Lineaments

**Fig. 4:**
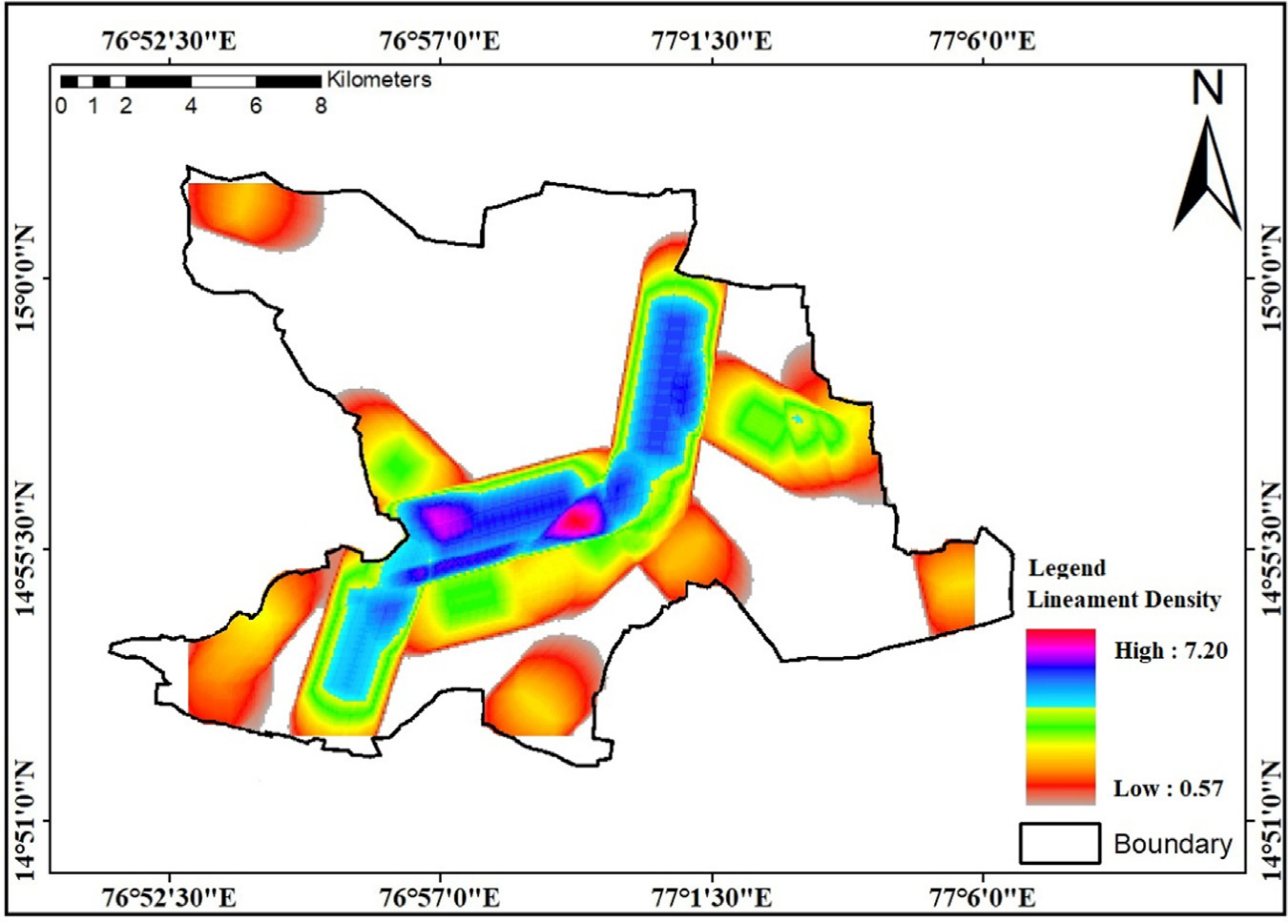
Lineament density map of the study area in km/km^2^.

In areas of semi-arid regions lineaments, fractures and joints are best conduits in movement and storage of groundwater [10]. Based on the lineament density factors, low density areas are having the higher runoff and high-density areas are having the higher rate of infiltration [11]. Considering the lineaments presence lineaments density calculate by way of the entire lengths of all lineaments segments of the study region which is divided with the aid of total area of the study region and outcome lineament density values shown Fig. 4.

### 2.3 Soil

**Fig. 5:**
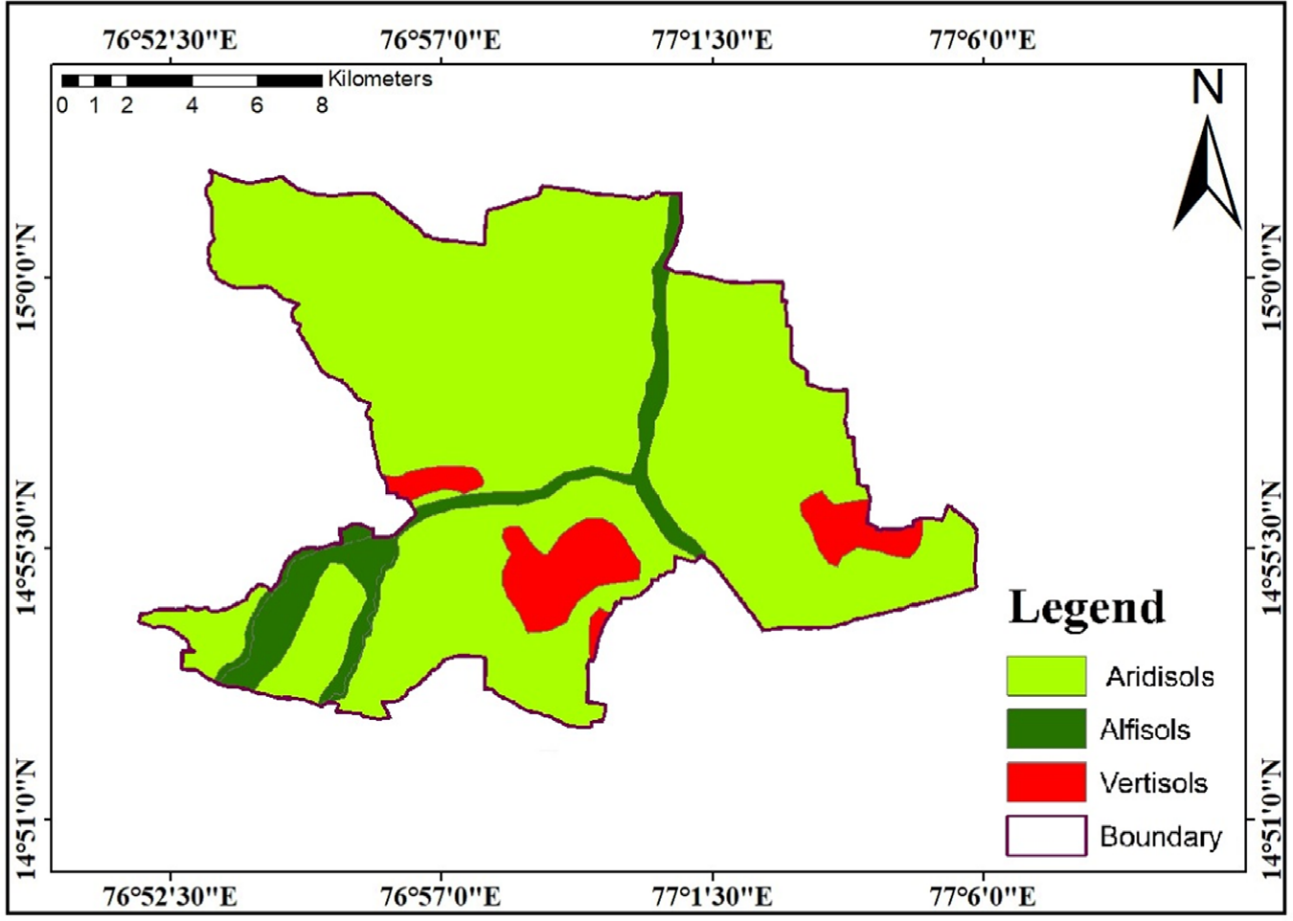
Soil map of the study area.

The Soil is most important element that plays a key role in recharging the ground water and its movement. The most of the study region blanketed by desertification due to environmental effects. The overall study region covered three types of soil classification such as aridisols, alfisols and vertisols which are shown in Fig. 5. The most of the study area region is included through aridisols. Alfisols depleted in calcium carbonate however enriched in Al and Fe bearing minerals exhibit well- developed contrasting soil horizons lies an area with huge growth of migrated horizon silicate clay. Vertisols are heavy clay soils that exhibit massive enlargement and contraction due to the presence or absence of moisture. They are frequent in areas that have shale parent material and heavy precipitation and Vertisols are mostly blanketed northeastern part of the study region.

### 2.4 Geology

**Fig. 6:**
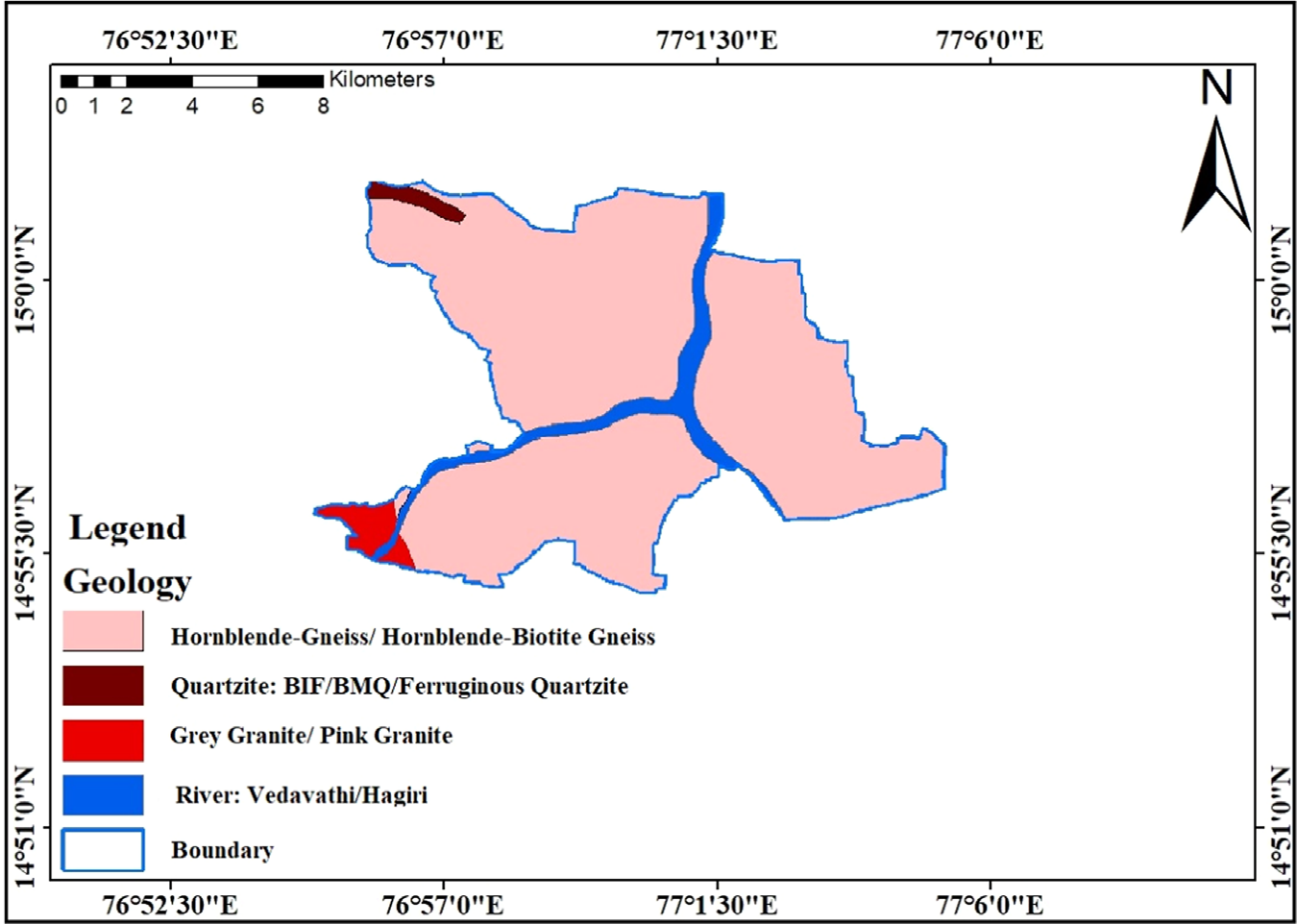
Geology map of the study area.

Groundwater occurs in permeable geologic formations known as aquifer and formations having structures that allow appreciable water to move through them under ordinary field conditions. The portion of a rock or soil not occupied by solid mineral count may also be occupied by groundwater. These areas are recognized as voids, interstices, pores, or pore spaces where interstices can act as groundwater conduits, they are of necessary importance to the study of groundwater. Typically, they are characterized by their size, shape, irregularity and distribution. Original interstices created by geologic approaches governing the origin of the geologic formation and are located in sedimentary and igneous rocks. On the other hand the secondary interstices developed after the rock was formed; For instance joints, fractures, solution openings and openings formed by plant life and animals. The main rock types existing in the study are usually felsic rocks, Hornblende-Gneiss, Hornblende- Biotite gneiss, grey and pink granites and Quartzites consist of BMQ/BIF ferruginous quartzites which are shown Fig. 6. The maximum study region overlaying Hornblende-gneiss with biotite gneiss. Some of the patches of ferruginous quartzite existing in the northwestern part of the study region and grey/pink granites are presented near components of southwestern part of the study area.

### 2.5 Geomorphology

**Fig. 7:**
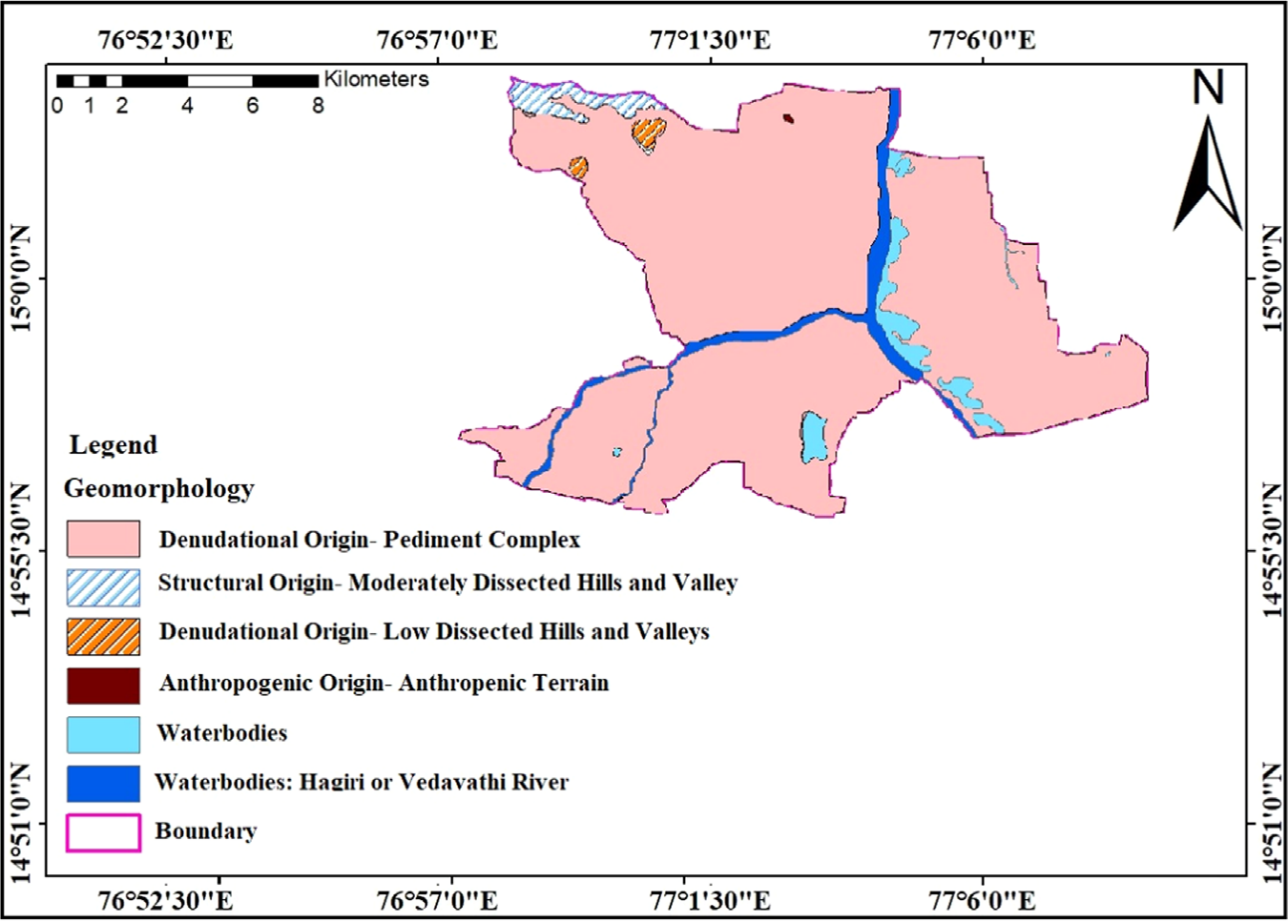
Geomorphology map of the study area.

Geomorphological aspects are more beneficial for delineation groundwater potential zones outcomes for the prevalence and movement of groundwater. Each geomorphic feature assists for occurrence and movement of groundwater. The exceptional geomorphic aspects are placed in the study region are Denudational origin-pediment complex, Denudational origin- Low dissected hills and valley, structural origin- Moderately dissected hills and valley, Anthropogenic origin and Water bodies have shown Fig. 7. The study region occupied by Denudational origin-pediment complex, this characterized good potential region in the study region due to low runoff. The structural origin- Moderately dissected hills and valley are the excessive infiltration of groundwater of the study region having low relief. The structural origin of the study region blanketed by secondary structures is good potential zones for the study area. The area covered by water canal, river and tanks these features are having property in excessive groundwater potential sector of the study area. Denudational origin-pediment complicated included in normally common study region in north-east to east-west. Structural foundation blanketed in the southwest section of the study area. The structural and Denudational pediment complicated having properly manageable zones of the study area.

### 2.6 Slope

**Fig. 8:**
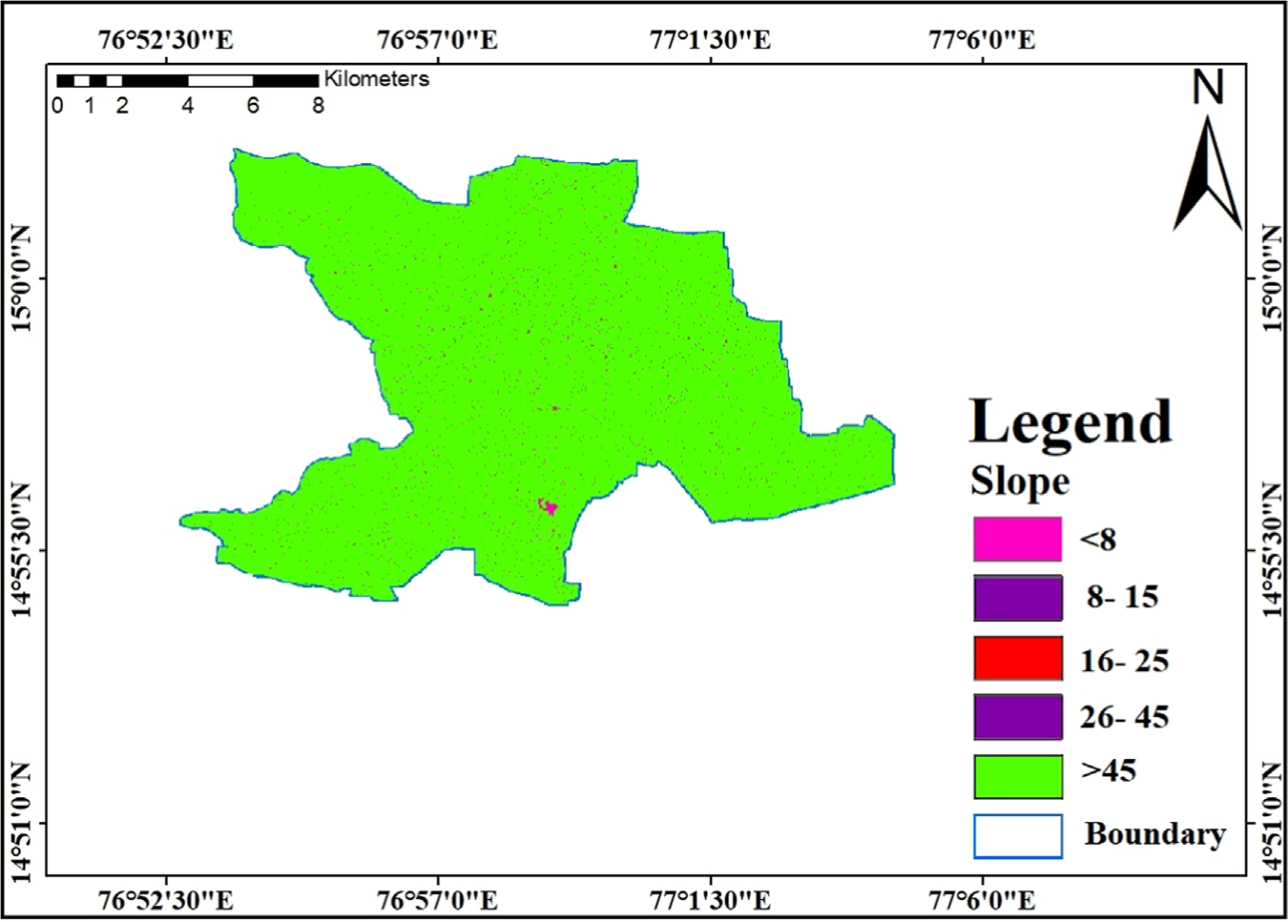
Slope map of the study area.

A Slope is additionally a necessary property for prevalence and recharging prerequisites of groundwater in a study area. Slope mentioned in degrees [12], [13], [14]. Considering on the slope factors the gentle slope, decreased will be the runoff and thus, higher is the groundwater recharge and steeper slope having increased runoff having lesser in groundwater recharge. The slope was estimated by using SRTM DEM data from the USGS website for the study region. In the region slope varies from 0° to greater than 45° which are shown in Fig. 8. The complete slope map is divided into 5 classes as follows the five classes in the study vicinity are nearly level to gentle slope (0– <8, 8- 15, 16–25), 26–2.45, more than 45°. Major Phase of the study region falls under nearly degree to gentle slope class (>45°).

### 2.7 Landuse/Landcover

**Fig. 9:**
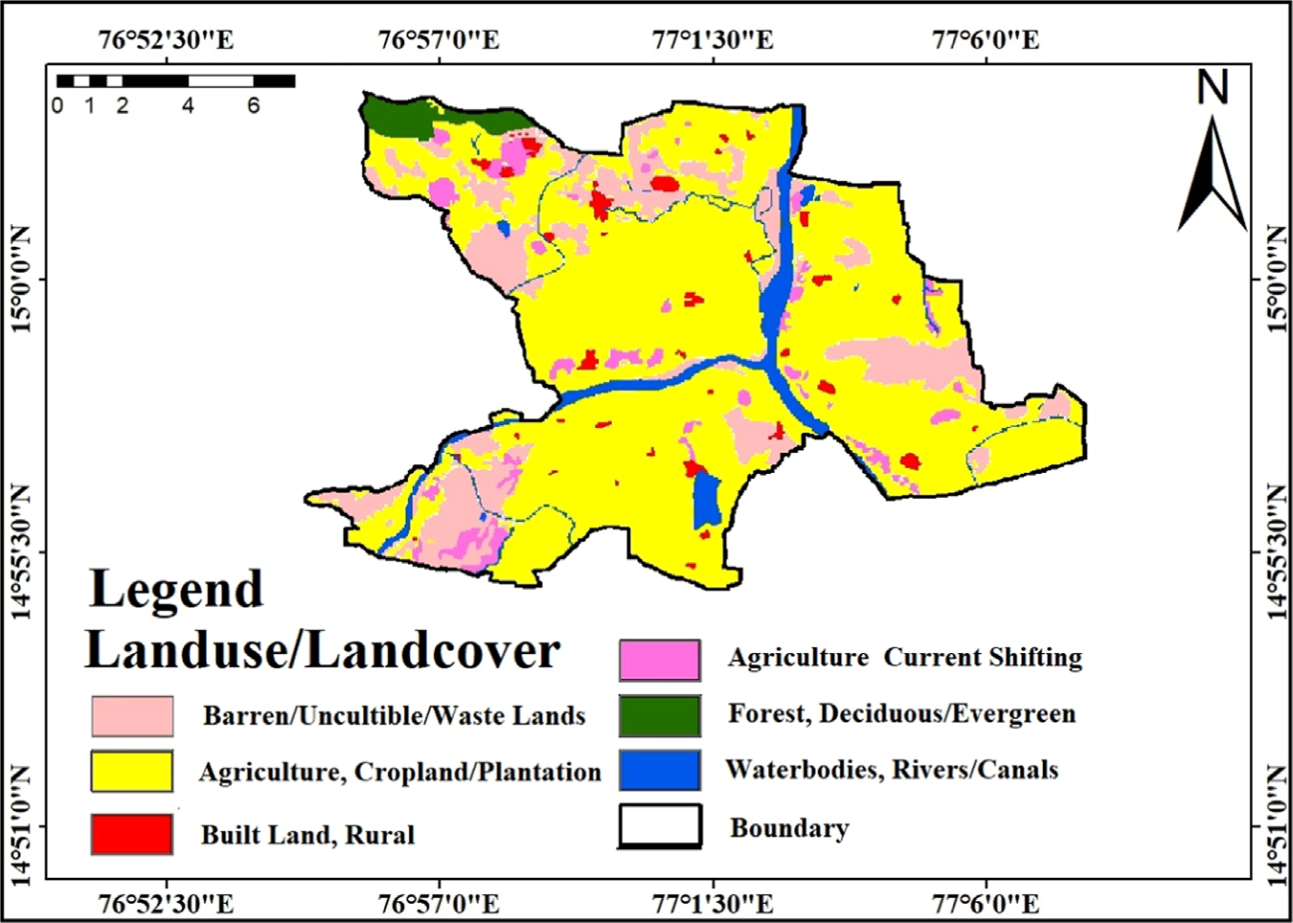
Landuse/landcover map of the study area.

Remote Sensing and GIS are essential technologies for evaluation and quantification of the spatial phenomenon which is in any other case not feasible to attempt through conventional mapping techniques [15]. Land use/cover aspects are planning and development of land and water resources the land use/cover classification analyzed by supervised classification technique with maximum likelihood algorithm used to apply in the ERDAS Imagine software. Supervised classification techniques used with Remote Sensing image data. This technique is based on the prospect that a pixel belongs to a specific class. The primary theory assumes that these possibilities are equivalent for all classes and that the input bands have everyday distributions. The various Landcover changes occurred in less time with higher accuracy by using geospatial technologies [16]. Considering signature classification categorized into unique features they are Agriculture land, Water bodies, Forest, Built-up land and Barren/uncultivable land shown in Fig. 9. These values are regrouped to produce a LU/LC map rank with normalized weights for artificial recharge sites.

### 2.8 Interrelationships between the factors of the artificial zones

The groundwater movement and occurrence in study region are distinctive aspects. The outcome of all elements need not be compatible in the area. Every parameter is assigned a weightage depending on its influence on the migration, occurrence and storage of ground water. The migration and occurrence of groundwater in a study region are managed by the aspects of all factors need no longer be the same in the area. In order to calculate the weights of the concerned factors, the outcomes of these on every different must be estimated [17]. The weightage of major and minor connection between two thematic layers assigned weights are 1 and 0.5. In hard rock semi-arid region of geomorphological manage is high balanced than geological control, so greater weightage is given to geomorphology than the geology. The weightage for artificial recharge is given in Table 1. The normalized Weights are assigned to distinctive thematic layers using AHP which consist of geology, geomorphology, lineament density, land use/land cover, soil, drainage density and slope provides the certain clue for the prevalence of groundwater. The pair wise evaluation for the seven layers had been given based on the assessment between the layers and their relative importance toward ground water potentialities and a 7×7 matrix is formed. On the basis of comparison matrix the following steps have been carried out to calculate the normalized weight. In steps1 each thematic layer of the column has been divided by way of their corresponding sum of the row to shape the relative weight matrix. In step2 the geometric imply was once obtained by means of averaging throughout the rows and normalized weight is once obtained by means of dividing each geometric mean thematic map with a sum of geometric suggest is shown in Table 2. In the current study, every parameter was assigned a rank which is depending on its impact on the storage and movement of groundwater. Each parameter is categorized into 5 zones from the artificial recharge point of view. The unique parameters in every aspect had been assigned information which is considering the relative importance of artificial recharge from 1 to 5 following their importance with reference to their influence on an identification of artificial recharge zones. In the ranking 1 denotes poor favorable zone, 2 denotes moderately favorable zone, 3 denotes moderate to good zone, 4 denotes good zone and 5 denotes excellent zone for identification of artificial recharge zones and artificial recharge sites.

**Table 1:** Weight influence for identifying artificial recharge site.

S. Nos.	Factors	Weightage
1	Geology	4.5
2	Lineament Density	3
3	Landuse/Landcover	4
4	Geomorphology	5
5	Soil	3.5
6	Drainage density	4
7	Slope	3

**Table 2:** Pair-wise comparison matrix for the seven themes and calculation of normalized weights by the analytic hierarchy process.

Theme	Theme							Geometric mean	Normalized weight
	GG	LD	LU/LC	GM	Soil	DD	Slope		
GG	4.5/4.5	4.5/3	4.5/4	4.5/5	4.5/3.5	4.5/4	4.5/3	0.17	0.17
LD	3/4.5	3/3	3/ 4	3/5	3/3.5	3/ 4	3/3	0.78	0.11
LU/LC	4/4.5	4/3	4/4	4/5	4/3.5	4/4	4/3	1.04	0.15
GM	5/4.5	5/3	5/4	5/5	5/3.5	5/4	5/3	1.30	0.19
Soil	3.5/4.5	3.5/3	3.5/4	3.5/5	3.5/3.5	3.5/4	3.5/3	0.91	0.13
DD	4/4.5	4/3	4/4	4/5	4/3.5	4/4	4/3	1.04	0.15
Slope	3/ 4.5	3/3	3/4	3/5	3/3.5	3/4	3/3	0.78	0.11

### 2.9 Suitable artificial recharge sites

**Fig. 10:**
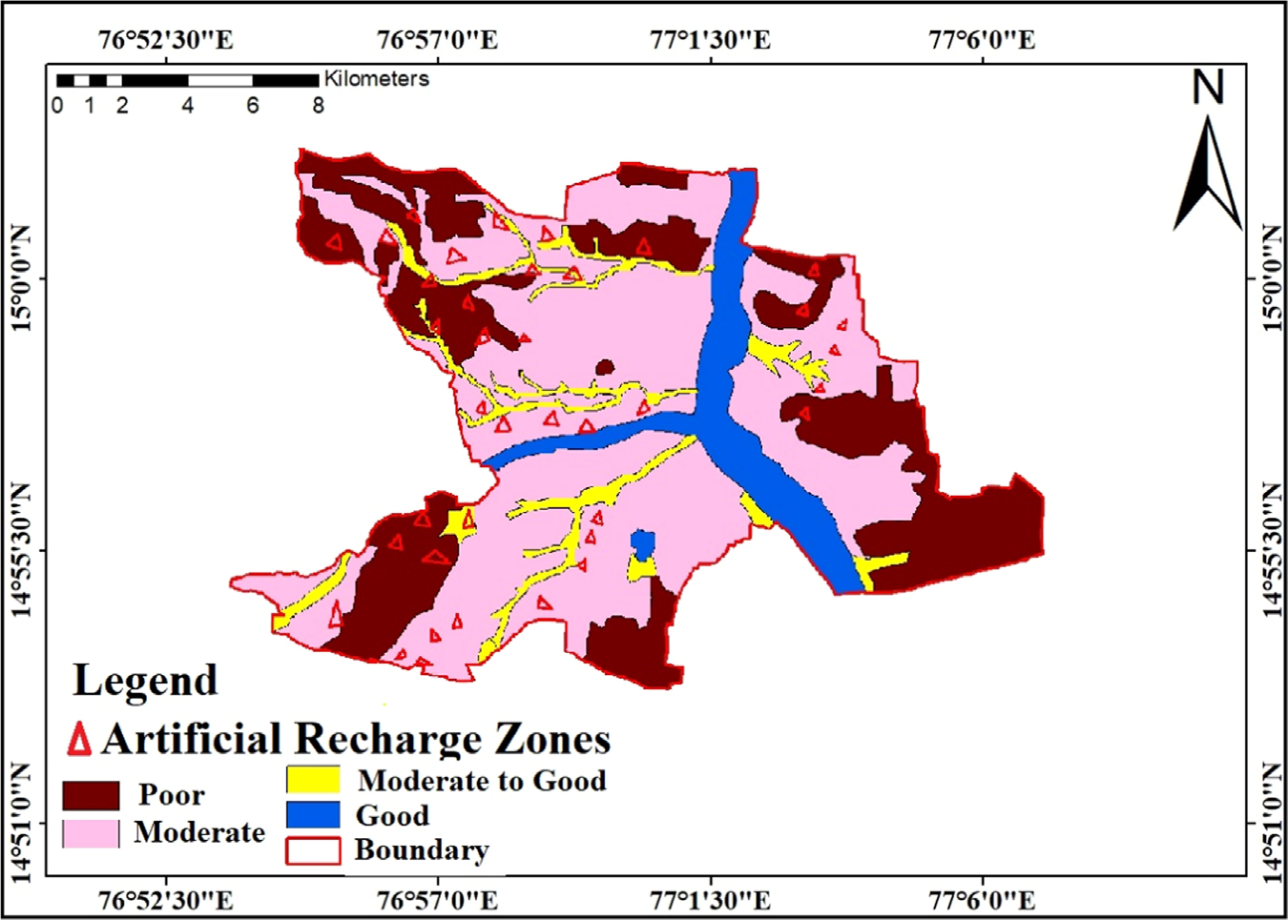
Artificial recharge sites.

In the present study, artificial recharge map is prepared by superimposing the lineament and drainage maps are relieved the favorable sites of the study region shown in Fig. 10. According to Fig. 10, 29 artificial recharge regions are recognized, of which 3 regions fall in the ׳great׳ recharge zone, 10 regions fall in the ׳moderately to great׳ recharge zone, 6 regions fall in the ׳moderate׳ recharge zone and 10 regions fall in ׳Poor׳ recharge zone. To the extent the artificial recharge structures are concerned, essentially check dams and permeation lakes are suggested at the distinguished destinations for artificial recharge in the study region. These structures are small scale structures that can be built across over lower order streams so as to improve infiltration into the subsurface formations.
