# Supplementary material for: Data on artificial recharge sites identified by geospatial tools in semi-arid region of Anantapur District, Andhra Pradesh, India
Source: Data Brief. 2018 Apr 21;19:462–74. doi: 10.1016/j.dib.2018.04.050 (PMC5997622; doi:10.1016/j.dib.2018.04.050)
Supplement: Supplementary file 1 — Supplementary material [file mmc1.doc]

**Conflict of Interest and Authorship Conformation Form**

- All authors have participated in conception and design, or analysis and interpretation of the data.
- This manuscript has not been submitted to, nor is under review at, another journal or other publishing venue.
- The authors have no affiliation with any organization with a direct or indirect financial interest in the subject matter discussed in the manuscript.
- The following authors have affiliations with organizations with direct or indirect financial interest in the subject matter discussed in the manuscript:

**Author’s name**  **Affiliation**

M .Rajasekhar Yogi Vemana University, Kadapa, Andhra Pradesh, India.

G.Sudarsana Raju Yogi Vemana University, Kadapa, Andhra Pradesh, India.

R.Siddi Raju Yogi Vemana University, Kadapa, Andhra Pradesh, India.

U.Imran Basha Sri Venkateswara University, Tirupati, Andhra Pradesh, India.


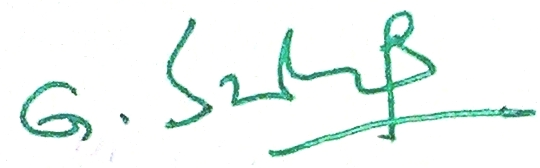


Corresponding Author Signature
